# Supplementary material for: TIMP-3 facilitates binding of target metalloproteinases to the endocytic receptor LRP-1 and promotes scavenging of MMP-1
Source: Sci Rep. 2020 Jul 21;10:12067. doi: 10.1038/s41598-020-69008-9 (PMC7374751; doi:10.1038/s41598-020-69008-9)
Supplement: Supplementary file 1 — Supplementary information [file 41598_2020_69008_MOESM1_ESM.docx]

**TIMP-3 facilitates binding of target metalloproteinases to the endocytic receptor LRP-1 and promotes scavenging of MMP-1.**


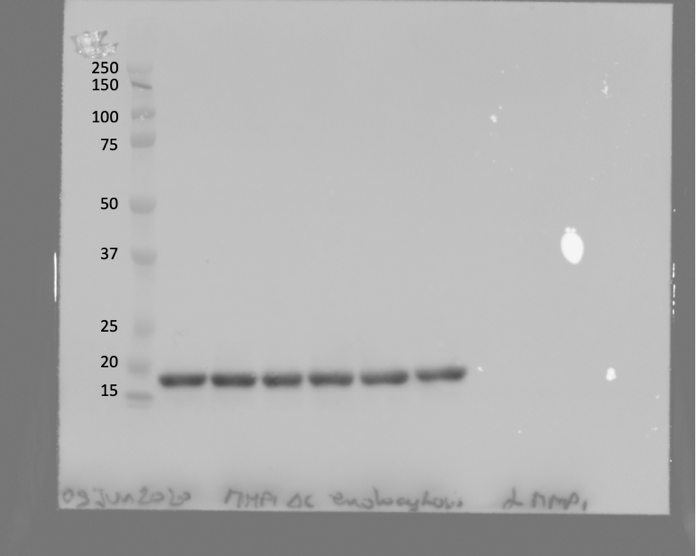
Anna P. Carreca^1^, Veronica Pravatà^2^, Matthew Markham^3^, Simone Bonelli^1^, Gillian Murphy^4^, Hideaki Nagase^5^, Linda Troeberg^3#^, Simone D. Scilabra^1#*^.

>MMP-1 ΔC


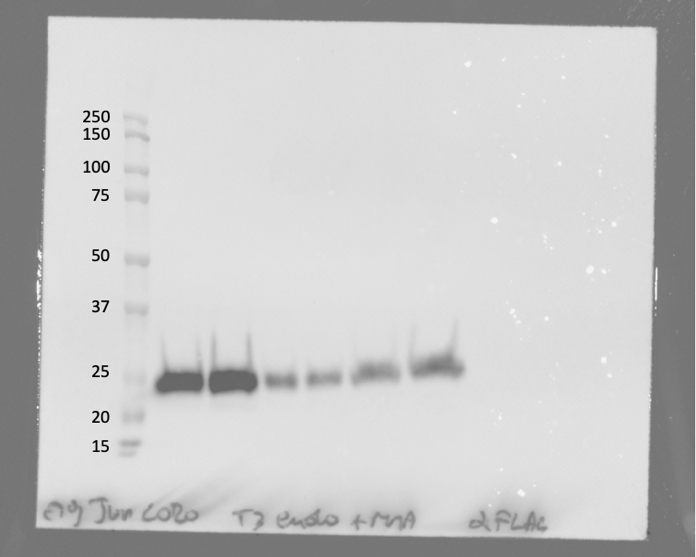


>TIMP-3

(in complex with MMP-1 ΔC)


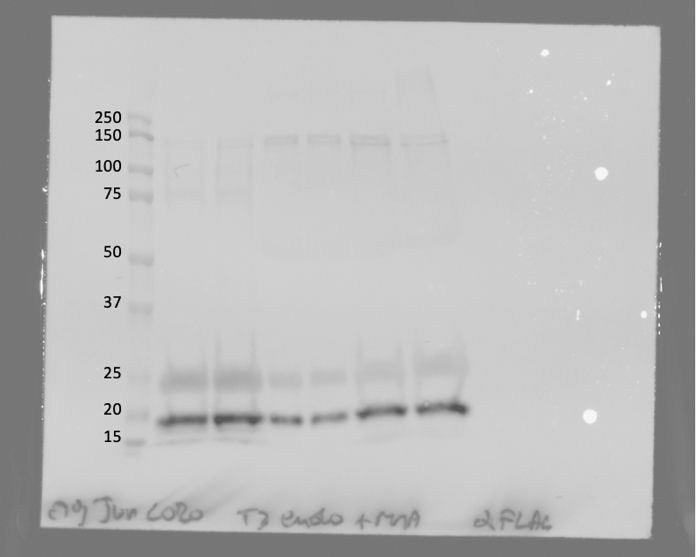


>MMP-1 ΔC

(in complex with TIMP-3)
